# The Role of Fecal *Fusobacterium nucleatum* and *pks^+^ Escherichia coli* as Early Diagnostic Markers of Colorectal Cancer

**DOI:** 10.1155/2021/1171239

**Published:** 2021-11-22

**Authors:** Kaixi Liu, Xinran Yang, Mi Zeng, Yumeng Yuan, Jianhong Sun, Ping He, Jiayu Sun, Qingdong Xie, Xiaolan Chang, Suwei Zhang, Xiang Chen, Leshan Cai, Yanxuan Xie, Xiaoyang Jiao

**Affiliations:** ^1^Departments of Clinical Laboratory, Shantou Central Hospital, Shantou, China; ^2^Departments of Clinical Laboratory, The First Affiliated Hospital of Shantou University Medical College, China; ^3^Medical College of Shantou University, Shantou, China; ^4^Departments of Clinical Pathology, Shantou Central Hospital, Shantou, China; ^5^Departments of Health Care Center, The First Affiliated Hospital of Shantou University Medical College, China

## Abstract

**Background:**

Accurate analysis of intestinal microbiota will facilitate establishment of an evaluating system for assessing colorectal cancer (CRC) risk and prognosis. This study evaluates the potential role of *Fusobacterium nucleatum* (*F. nucleatum*) and *Escherichia coli* with a *pks* gene (*pks^+^ E. coli*) in early CRC diagnosis.

**Methods:**

We recruited 139 patients, including CRC (*n* = 60), colorectal adenomatous polyposis (CAP) (*n* = 37), and healthy individuals (*n* = 42) based on their colonoscopy examinations. We collected stool and serum samples from the participants and measured the relative abundance of *F. nucleatum* and *pks^+^ E. coli* in fecal samples by quantitative PCR. Receiver operating characteristic curve (ROC) analyses were used to analyze the diagnostic value of single or combined biomarkers.

**Results:**

Fecal *F. nucleatum* and *pks^+^ E. coli* levels were higher in the CRC group in either the CAP group or healthy controls (*P* = 0.02; 0.01). There was no statistical difference in the distribution of *F. nucleatum* and *pks^+^ E. coli* in patients with different tumor sites (*P* > 0.05). The combination of *F. nucleatum*+*pks^+^ E. coli*+CEA+CA19-9+FOBT was chosen as the optimal panel in differentiating both CRC and CAP from the controls. The combination of *F. nucleatum*, *pks^+^ E. coli*, and FOBT improved diagnostic efficiency. However, there was difficulty in differentiating CRC from CAP.

**Conclusion:**

Our results suggested that combining bacterial markers with conventional tumor markers improves the diagnostic efficiency for noninvasive diagnosis of CRC.

## 1. Introduction

Colorectal cancer (CRC) is the third most common malignancy and the fourth leading cause of cancer-related deaths worldwide [1]. Although surgery and subsequent chemotherapy have made significant progress in CRC treatment, its mortality remains very high. The prognosis of CRC is highly dependent on the tumor stage, with patients at stage I having an excellent prognosis with 5 − year survival rates > 90% following surgical resection. Once the tumor cells have metastasized, the outcome is abysmal. The 5-year survival rates for stage IV patients are less than 10% [2]. Thus, early diagnosis will enable prompt tumor treatment to dramatically reduce CRC mortality [3].

The current diagnostic methods for CRC include invasive and noninvasive techniques. The fecal occult blood tests (FOBTs) are the primary screening methods for CRC and have advantages of secure and noninvasive sample collection. The guaiac fecal occult blood test can be quickly done unsupervised at home with a sample collection kit [4]. Therefore, it is widely accepted by patients and suitable for large-scale population screening. However, the main challenge comes from its low sensitivity and specificity. FOBTs fail to detect 50% of asymptomatic CRC [5]. More importantly, the test has dietary or time restrictions as certain foods in the diet may cause false-positive results. Besides CRC, some diseases, including ulcers, hemorrhoids, adenoma, or inflammation, can also cause gastrointestinal bleeding and give a positive result in FOBTs, resulting in misdiagnosis. Colonoscopy can improve the detection rate of CRC due to it is high sensitivity and specificity in the differential diagnosis of hemorrhoids, adenomas, and CRC. However, the disadvantages of colonoscopy include high costs, a higher risk of complications such as perforation and bleeding, discomfort from bowel preparation or embarrassment, and fear of the procedure, making colonoscopy hard to perform on the general population [6, 7]. Blood tumor markers, including carcinoembryonic antigen (CEA), carbohydrate antigen 19-9 (CA19-9), and CA125, are currently the main tumor biomarkers for CRC, which play significant roles in CRC screening, diagnosis, and treatment monitoring. However, inadequate sensitivity or specificity limits their application in discriminating high-risk CRC groups, in the early stage of cancer, from the general population [7]. Recent progress in proteomics has opened new avenues for cancer-related marker discoveries, presenting an opportunity to develop highly sensitive diagnostic tools for the early detection of cancers. Unfortunately, none of the serum proteins are recommended for early diagnosis. Currently, the idea of early CRC screening is not widely accepted by the public despite their apparent advantages, and participation rates in screening programs remain too low, with approximately 40% of the recommended population, recommended for undergoing CRC testing, not complying.

CRC is a complex disease that is influenced by both genetic and environmental factors. Accumulating evidence suggests that gut microbiota or its metabolites may be proximate environmental modifiers of risk for CRC [8, 9]. The human gut is the host to roughly a thousand different bacterial species, containing beneficial commensal bacteria and potentially pathogenic bacteria. CRC carcinogenesis may result from dysbiosis in the colonic microbiota with an increased proportion of certain bacteria whose metabolism produces cytotoxic or genotoxic compounds that cause DNA damage either through the production of free radicals or through abnormal activation of resident immune cells [10]. Once the intestinal balance is damaged, numerous intestinal diseases could result, including inflammatory bowel diseases (IBD) and colorectal neoplasms [11, 12]. Additionally, specific intestinal bacterial agents may be significant factors contributing to the accumulation of mutations that often manifest during cancer cell differentiation and development in the gut. *Fusobacterium nucleatum* (*F. nucleatum*) has been pointed out as initial triggers in CRC development [13]. *F. nucleatum* elicits a proinflammatory microenvironment around the tumor, driving tumor formation and progression [14]. The amount of tissue *F. nucleatum* is inversely associated with CD3^+^ T-cell density in CRC tissue [15] and the Fap2 outer-surface protein of *F. nucleatum* binds and activates the human inhibitory receptor TIGIT, which is expressed by T and natural killer (NK) cells, and inhibits antitumor immunity [16]. The prognostic role of *F. nucleatum* may be different in diseases. *F. nucleatum*-high is associated with poor prognosis in metastatic CRC but not in stage III or high-risk stage II patients [17]. In addition to *F. nucleatum*, *Escherichia coli (E. coli)*, *Enterococcus faecalis*, *Streptococcus gallolyticus*, and *Enterotoxigenic Bacteroides fragilis* are candidate microorganisms that are closely associated with CRC carcinogenesis [13]. Pathogenic *E. coli* has different types. Cyclomodulin-producing *E. coli* (CPEC) has been associated with CRC [18]. Most CPEC strains harbor a colibactin-encoding polyketide synthase (pks) pathogenicity island, and these CoPEC strains are more prevalent in aggressive CRC tumors [18–20]. Animal studies showed that mucus degradation, enabling increased *pks^+^ E. coli* adherence, induces increased colonic epithelial cell DNA damage [21]. In addition, CoPEC strains induce cellular senescence associated with the production of growth factors, leading to an increase in tumor growth in chemically induced, colitis-associated CRC models [22]. Given the roles of *F. nucleatum* and *E. coli* in CRC carcinogenesis, they could serve as potential biomarkers to reflect pathogenesis and disease status.

Fecal-luminal microbiota can be acquired easily by collecting feces. Therefore, some large-scale studies, including some fundamental studies, such as the MeTaHIT cohort and Human Microbiome Project, are investigating human gut microbiota based on the fecal-luminal microbiota [23, 24]. Owing to the low sensitivity of FOBT, tumor markers, and the limitation of colonoscopy in early diagnosis, the discovery of new markers with high sensitivity and specificity would be a major step in the early diagnosis of CRC. Based on our previous study [25], a higher abundance of *F. nucleatum and E. coli* were observed in CRC patients compared with normal individuals. Therefore, in this study, we investigated the ability of fecal *F. nucleatum* and *E. coli* to serve as biomarkers for early CRC diagnosis.

## 2. Methods

This trial was conducted in accordance with the Declaration of Helsinki (as revised in 2013). The ethics committee of Shantou University Medical College and the Shantou Central Hospital approved this study. Written informed consent was obtained from all patients. Before the procedure, patients were informed that their information would include age, gender, WBC, RBC, Hb, PLT, CEA, and stool samples and would be collected for scientific research.

### 2.1. Patients

A total of 139 patients recruited from January 2019 to December 2019 in Shantou Central Hospital were included in this study and included CRC and CAP patients and normal controls. The patients with CRC and CAP were selected based on the following criteria: CRC patients were TNM stages I-IV, and clinical and histopathologic staging at diagnosis was determined in all patients by combining histopathologic findings with surgical records and perioperative imaging. Control subjects were selected randomly from healthy individuals undergoing colonoscopy screening and had normal colorectal mucosae. We excluded patients who used antibiotics or prebiotics used before the sample collection. Stool and serum were collected from CRC patients at diagnosis and before first-line chemotherapy. When samples arrived at the laboratory, they were homogenized or centrifuged and stored at -80°C. In addition, patient demographic data, including age, gender, pathological type, and TNM staging, were recorded.

### 2.2. Measurement of *F. nucleatum* and *pks^+^ E. coli*

#### 2.2.1. DNA Extraction from Stool

According to the manufacturer's instructions, DNA was extracted from all fecal samples using a nucleic acid extract mini kit (magnetic bead method) (TIAN LONG NP968, Xi'an China). A NanoDrop 2000 spectrophotometer (Thermo Fisher Scientific, Waltham, MA, USA) was used for measurements of concentration and purity of the extracted DNA.

#### 2.2.2. Real-Time Quantitative PCR (qPCR)

Oligonucleotide primers were designed on Primer 5.0 and synthesized by Sango Biotech Co. (Shanghai, China) (Table 1). The target microbiomes and reference genes (16srRNAs) were quantified on a Roche LightCycler480 (Basel, Switzerland). PCR reactions included 30 ng DNA, 2×Talent qPCR Pre-Mix (SYBR Green) (TIANGEN, Beijing, China), 10 *μ*M primer mix, and RNase-free water in 25 *μ*l. All reactions were detected under the following conditions: one cycle of 95°C for 5 min, 45 cycles of 95°C for 10 sec, and 57°C for 30 sec. The results were analyzed using the Roche480 2.0 software. Total bacterial DNA determined by 16srRNA qPCR was used to normalize the target genes in fecal samples. All qPCR reactions were performed in duplicate. Relative quantities of *F. nucleatum* and *pks^+^ E. coli* in each stool sample were determined by the 2^-△Ct^ method, using 16srRNA gene as a reference gene: △Ct = Ct_Fn/E.coli with pks gene_ − Ct_16SrRNA_.

### 2.3. Measurement of Blood Tumor Markers and Other Biochemical Parameters

Three-milliliter venous blood samples were drawn from patients after an overnight fast. CEA and CA19-9 were measured with the chemiluminescence method using an automated system (Beckman I800). The blood routine index was measured by an automated system (Sysmex XN-2000). FOBT was measured by chemical methods (BASO, Zhuhai, China) according to the manufacture's instruction.

### 2.4. Statistical Analyses

For statistical comparison of means between independent groups, one-way analysis of variance (ANOVA) was performed to test differences in both *F. nucleatum* and *pks^+^ E. coli* between the CRC, CAP, and control groups, followed by the least significant difference post hoc test. To estimate the diagnostic value of single or combined biomarkers, we used receiver operating characteristic curve (ROC) analyses. Performance of the markers was analyzed by calculating the area under the receiver-operating characteristic curve (AUC) and compared using Delong's test. The sensitivities and specificities were compared using the McNemar paired comparison test. All statistical analyses were carried out using SPSS 20.0. *P* values less than 0.05 were considered statistically significant.

## 3. Results

One hundred thirty-nine patients were divided into three groups, based on the colonoscopy and pathological results: CRC (*n* = 60, age: 64.11 ± 11.14), CAP (*n* = 37, age: 66.50 ± 3.53), and control group (*n* = 42, age: 61.00 ± 7.90). The proportion of females in each group was 46.7%, 48.6%, and 26%, respectively (Table 2). The colonoscopy examinations detected that tumor position, including sigmoid colon, cecum, rectosigmoid junction, rectum, ascending colon, transverse colon, and descending colon. Tumor markers CEA and CA19-9 tended to be higher in CRC, but the difference did not reach statistical significance. However, the FOBT in CRC was significantly higher than those in the CAP group (*P* < 0.05).

### 3.1. Levels of *F. nucleatum* and *pks^+^ E. coli* CRC Patients

**Figure 1:**
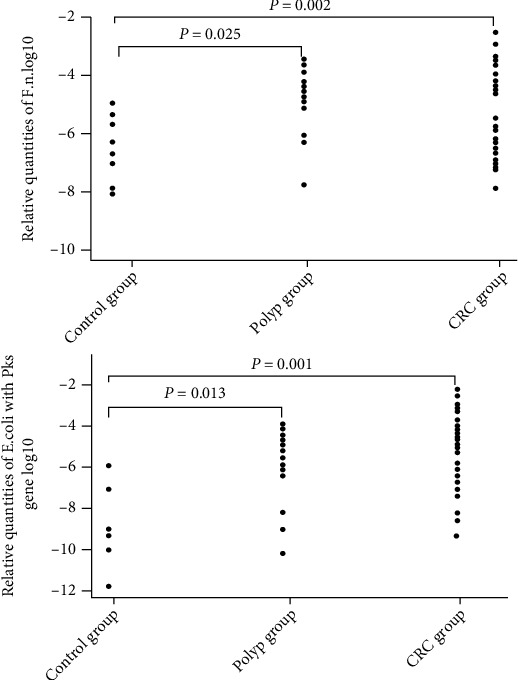
Relative quantities of *F. nucleatum* and *pks^+^ E. coli* in patients with CRC, CAP, and the controls.

The abundance distribution of *F. nucleatum* was (2.31 ± 1.01) × 10^−4^ in the CRC group, (1.98 ± 4.11) × 10^−4^ in the CAP group, and (5.34 ± 1.96) × 10^−7^ in the control group. There was no significant difference between CRC and CAP groups, but significantly higher levels of *F. nucleatum* were observed in the CRC and CAP groups compared to the control group (*F* = 5.221, *P* = 0.008) (Figure 1(a)). The abundance of *pks^+^ E. coli* was (4.39 ± 2.13) × 10^−4^ in the CRC group, (1.34 ± 5.68) × 10^−5^ in the CAP group, and (2.11 ± 7.12) × 10^−9^ in the control group. Significantly higher levels of *pks^+^ E. coli* were observed in the CRC group compared to the CAP and control groups, and higher levels were also found in the CAP group compared with the control group (*P* < 0.05) (Figure 1(b)).

**Figure 2:**
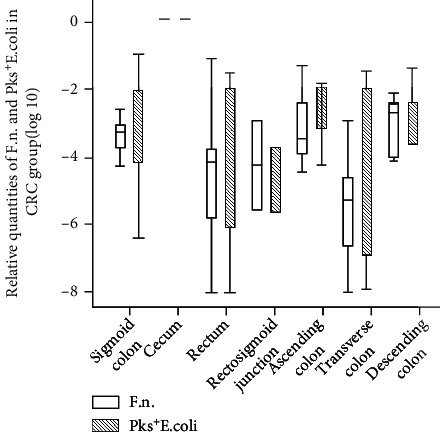
Relative quantities of *F. nucleatum* and *pks^+^ E. coli* in CRC patients with various tumor locations.

Further studies were conducted to analyze the abundance of *F. nucleatum* and *pks^+^ E. coli* in CRC patients with tumors located in seven different intestine sites. The abundance of *F. nucleatum* was (3.32 ± 1.86) × 10^−3^ in the sigmoid colon, (4.01 ± 3.89) × 10^−4^ in the rectum, (3.23 ± 2.01) × 10^−5^ in the rectosigmoid junction, (2.89 ± 3.01) × 10^−3^ in the ascending colon, (3.51 ± 2.87) × 10^−4^ in the transverse colon, and (2.11 ± 1.67) × 10^−4^ in the descending colon. The abundance of *pks^+^ E. coli* was (3.41 ± 1.86) × 10^−3^ in the sigmoid colon, (4.15 ± 3.71) × 10^−4^ in the rectum, (3.41 ± 1.86) × 10^−5^ in the rectosigmoid junction, (3.45 ± 2.92) × 10^−3^ in the ascending colon, (4.68 ± 3.55) × 10^−4^ in the transverse colon, and (3.82 ± 1.53) × 10^−4^ in the descending colon. There was no statistically significant difference in the distribution of *F. nucleatum* and *pks^+^ E. coli* in patients with tumors at different sites (*F* = 0.813, *P* = 0.67, and *F* = 2.602, *P* = 0.144, respectively) (Figure 2).

**Figure 3:**
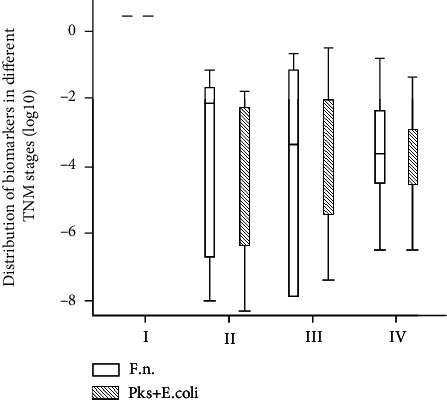
Relative quantities of *F. nucleatum* and *pks^+^ E. coli* in CRC patients at various TNM stages.

We also investigated the distribution of the abundance of *F. nucleatum* and *pks^+^ E. coli* at different TNM stages in the CRC group. The results showed that *F. nucleatum* and *pks^+^ E. coli* were not detected in CRC stage I patients. The abundance of the *F. nucleatum* in stage II was (2.81 ± 4.34) × 10^−4^, (2.48 ± 5.13) × 10^−4^ in stage III, and (2.32 ± 1.05) × 10^−4^ in stage IV. There was no statistically difference found between various cancer stages (*F* = 3.487, *P* = 0.246). Similarly, the abundance of *pks^+^ E. coli* was (2.45 ± 5.34) × 10^−4^ in stage II, (2.42 ± 4.01) × 10^−4^ in stage III, and (2.36 ± 1.84) × 10^−4^ in stage IV, indicating no significant cancer stage-dependent differences of *pks^+^ E. coli* abundance (*F* = 0.912, *P* = 0.617) (Figure 3).

### 3.2. Diagnostic Value of *F. nucleatum* and *pks^+^ E. coli*

**Figure 4:**
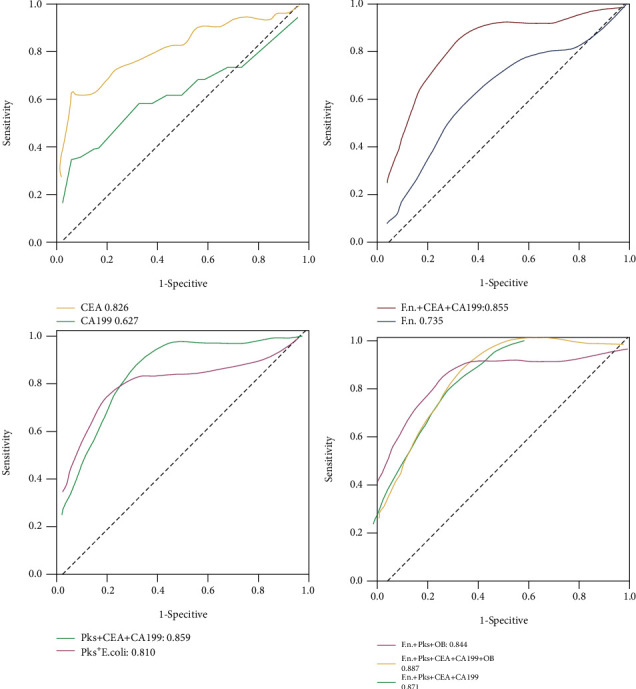
Diagnostic efficiency of *F. nucleatum*, *pks^+^ E. coli*, CEA, CA19-9, and FOBT in differentiating the CRC from normal controls.

To estimate the diagnostic value of single or combined biomarkers, we used receiver operating characteristic curve (ROC) analyses. We first evaluated the performance of the single markers, including *F. nucleatum* and *pks^+^ E. coli*, as well as the conventional tumor markers CEA and CA19-9, to serve as individual markers in differentiating CRC from the controls (Table 3). The AUCs (from high to low) were CEA (0.826 (95% CI 0.73 to 0.91)), *pks^+^ E. coli* (0.810 (95% CI 0.67 to 0.96)), *F. nucleatum* (0.735 (95% CI 0.59 to 0.87)), and CA19-9 (0.627 (95% CI 0.51 to 0.75)). In distinguishing between CAP and normal individuals, the highest AUC was observed for *pks^+^ E. coli* 0.818 (95% CI (0.64 to 0.98)), followed by *F. nucleatum* (0.741 (95% CI 0.56 to 0.91)), CA19-9 (0.764 (95% CI 0.64 to 0.88)), and CEA (0.710 (95% CI 0.57 to 0.84)), respectively. However, in distinguishing between CRC and CAP, the diagnostic value of all indicators was not very good. The AUCs for CEA, CA19-9, *F. nucleatum*, and *pks^+^ E*. *coli* were 0.684 (0.51, 0.85), 0.440 (0.26, 0.61), 0.514 (0.33, 0.69), and 0.389 (0.21, 0.56), respectively (Table 3 and Figure 4).

**Figure 5:**
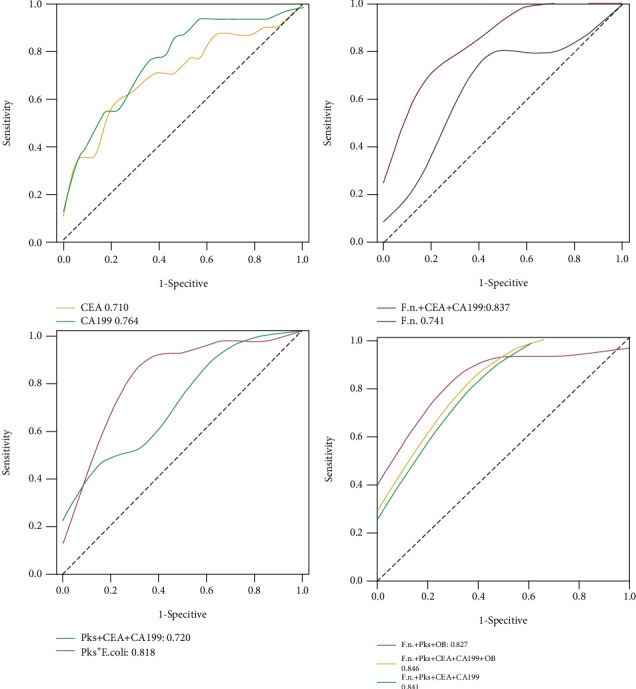
Diagnostic efficiency of *F. nucleatum*, *pks^+^ E. coli*, CEA, CA19-9, and FOBT in differentiating the CAP from normal controls.

**Figure 6:**
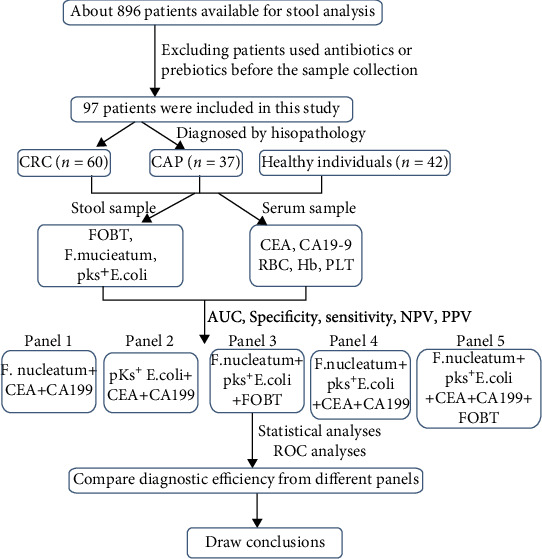
Schematic diagram of the experimental design from the sample collection to statistical analyses.

Better diagnostic efficiency was obtained from panels of markers than from a single biomarker. The combinations of *F. nucleatum*+CEA+CA19-9, *pks^+^ E. coli*+CEA+CA19-9, *F. nucleatum*+*pks^+^ E. coli*+FOBT, *F. nucleatum*+*pks^+^ E. coli*+CEA+CA19-9, and *F. nucleatum*+*pks^+^ E. coli*+CEA+CA19-9+FOBT showed a higher AUC compared with a single marker, all AUCs of which were greater than 0.8. The panel of *F. nucleatum*+*pks^+^ E. coli*+CEA+CA19-9+FOBT (panel 5) was chosen as optimal panel in differentiating CRC from the controls, with an AUC of 0.887 (95% CI 0.68 to 1.0), sensitivity of 75.0%, specificity of 98.1%, NPV of 0.98, and PPV of 0.77. *F. nucleatum*+*pks^+^ E. coli*+FOBT (panel 3) also had a high AUC (0.844 (95% CI 0.71 to 0.97)). The advantage of this panel was that the specimen only needed feces rather than blood. In differentiating CAP from controls, panel 5 had the highest AUC (0.846 (95% CI 0.57 to 1.0)), with a sensitivity of 66.7% and specificity of 90.3%, similar to the diagnostic efficiency of panel 3. However, there remained a difficulty in differentiating CRC vs. CAP. Panel 3 had the highest AUC: (0.560 (95% CI 0.38 to 0.73)), with a sensitivity of 80.6% and a specificity of 35.7% (Table 3 and Figure 5). These results suggested that adding the bacterial markers with conventional tumor markers improves the diagnostic efficiency for the noninvasive diagnosis of CRC (Figure 6).

## 4. Conclusions

Current knowledge on biomarkers with high efficiency for early diagnosis of CRC is limited, especially for highly desirable noninvasive testing. Extensive data confirm that several bacteria are involved in CRC carcinogenesis [26], with microbiota dysbiosis not only contributing to the malignant progression of cancer but also being crucial for the therapeutic efficacy of some anticancer drugs [7].

*F. nucleatum* plays a crucial role in CRC carcinogenesis and is involved with CRC recurrence and resistance to chemotherapy by activating the autophagy pathway [27]. Enrichment of *F. nucleatum* has been identified in CRC patients and is associated with worse outcomes [28, 29]. CRC tissues have a higher abundance of *F. nucleatum* and *B. fragilis* bacteria than normal tissues in Iranian patients, and it has been recommended that the role of CRC-associated bacteria in CRC be further investigated in vivo and in vitro [30]. As it can be detected in both CRC tissues and feces of patients with CRC [31], *F. nucleatum* could serve as a potential marker for diagnosing patients with CRC. For tumor screening, it is not convenient to obtain tissue specimens, but feces samples are more feasible [32]. In our study, fecal *F. nucleatum* was higher in CRC patients than that in normal individuals. However, there was no significant difference in *F. nucleatum* between in CRC and CAP, and no significant difference in *F. nucleatum* level was found in CRC patients at various TNM stages. A previous study also showed that high enrichment of *F. nucleatum* occurred in CRC tissues, but there was no difference in the degree of *F. nucleatum* enrichment between adenomas and advanced adenomas, and there is no significant difference in adenoma versus normal tissues [33]. There is disagreement about the relationship between *F. nucleatum* and colorectal adenoma [26]. The AUC of 0.735 (95% CI 0.59 to 0.87), with a sensitivity of 69.2%, and a specificity of 73.9% was obtained for the prognostic ability of *F. nucleatum*. Our results indicate that the diagnostic performance of fecal *F. nucleatum* was not optimal among all CRC tumor markers. Therefore, use of *F. nucleatum* alone may not provide enough diagnostic accuracy for early detection of CRC.

*E. coli* is commonly isolated from both CRC patients and healthy controls. However, more pathogenic strains are isolated from CRC patients than healthy individuals [34]. That *E. coli* occurs in patients with CRC and inflammatory bowel disease suggests an active involvement of enterobacterial toxins in tumorigenesis [2]. The genomic polyketide synthetase (pks) island is responsible for the expression of peptide-polyketide hybrid genotoxic-cyclomodulin (a nonribosomal peptide synthetase and pks) referred to as colibactin (clb). *pks^+^ E. coli* is closely associated with inflammatory bowel disease and sporadic CRC [34–36], and CRC patients more frequently harbor *pks^+^ E. coli* strains in their colonic mucosa than noncancerous patients [37]. *pks^+^ E. coli* might play a role in the initiation and promotion of carcinogenesis [37], but the potential utility of *E. coli* in detecting colorectal neoplasia remains underexplored. In this study, the abundance of fecal *pks^+^ E. coli* in patients with CRC and CAP was significantly higher than the normal population. Moreover, a high AUC (0.810 (95% CI 0.67 to 0.96)) and high sensitivity (93.3%) were found for *pks^+^ E. coli* in differentiating CRC from the controls. Moreover, *pks^+^ E. coli* had the highest AUC and high sensitivity (90.9%) in distinguishing between CAP and normal individuals. Sensitivity is the most important characteristic of a screening test, as its primary role is to identify samples for further diagnostic testing [38]. Thus, fecal *pks^+^ E. coli* could be a potential diagnostic marker for early CRC detection.

Currently, screening and early diagnosis of CRC still rely on conventional tumor markers, including FOBT, CEA, and CA19-9. The fecal immunochemical test (FIT) is the most used noninvasive test, but has low sensitivity for CRC and is not sensitive to adenoma. As there is disagreement about the diagnostic effectiveness that exists between conventional tumor markers and microbial markers, and the diagnostic efficacy of a single marker is not very high, we characterized combinations of the two types of markers to determine whether diagnostic efficiency could be increased. Results indicated that better diagnostic efficiency could be achieved compared to a single biomarker. All AUCs of the combinations were above 0.8, with the combination of *F. nucleatum*+*pks^+^ E. coli*+CEA+CA19-9+FOBT showed the highest AUC, with high sensitivity and specificity, in differentiating CRC from the controls. The panel of *F. nucleatum*+*pks^+^ E. coli*+FOBT also had a high AUC, but more importantly, the advantage of this panel is that specimens only require feces but not blood. Therefore, detection of *F. nucleatum* and *pks^+^ E. coli* in feces by qPCR, combined with FOBT, could increase the detection rate of CRC from normal individuals. In differentiating CAP from the control, the panel of *F. nucleatum*+*pks^+^ E. coli*+CEA+CA19-9+FOBT had the highest AUC (0.846), with a sensitivity of 66.7% and a specificity of 90.3%, respectively. Fecal *F. nucleatum* and *pks^+^ E. coli* combined with CA19-9 and CEA are more sensitive than only CA19-9 and CEA in screening for early-stage CRC, suggesting that fecal *F. nucleatum* and *pks^+^ E. coli* may be potential markers for the diagnosis of early-stage CRC.

It is now emerging that specific bacteria are implicated in the risk of CRC [39]. With the discovery of the vital role of the microbiome in CRC carcinogenesis and treatment, the use of microbial flora as tumor markers shows promise. Accurate analysis of the intestinal microbiota will facilitate establishing an evaluating system for assessing CRC risk and prognosis. Our results suggested that combining bacterial markers with conventional tumor markers will improve the diagnostic capability of noninvasive diagnosis of CRC, and stool-based bacteria could serve as noninvasive diagnostic biomarkers for CRC [40, 41]. Microbial markers may represent an essential strategy for CRC detection in the future to screen patients for “high-risk” microbial patterns and identify candidates for further diagnostic procedures such as colonoscopy.

## Figures and Tables

**Table 1:** Primers and probes used in this study.

	Primer sequence 5′-3′	Temperature (°C)	Amplicon size
F. *nucleatum*	F: CAACCATTACTTTAACTCTACCATGTTCA R: TACTGAGGGAGATTATGRAAAAARC	57	105 bp
pks^+^*E. coli*	F: TCACTGTCGTCCCTTTGACG R: TAATCGGATCGCCTGACAGC	58	146 bp
16srRNA	341-F: CCTACGGGNGGCWGCAG 805-R: GACTACHVGGGTATCTAATCC	57	464 bp

F: forward primer; R: reverse primer.

**Table 2:** General characteristic of patients and controls.

	Control	CAP	CRC
*N* (male/female)	42 (31/11)	37 (19/18)	60 (32/28)
Age	61.00 ± 7.90	66.50 ± 3.53	64.11 ± 11.14
WBC	4.32 ± 2.21	4.89 ± 3.05	5.04 ± 3.11
PLT	259 ± 31.12	266 ± 28.32	254 ± 30.01
RBC	4.49 ± 2.21	4.22 ± 3.01	4.18 ± 3.55
Hb	125 ± 11.31	122 ± 17.64	118 ± 15.13
CEA	2.01 ± 2.21	3.89 ± 3.33	4.99 ± 6.25
CA19-9	2.01 ± 2.21	3.89 ± 3.33	4.99 ± 6.25
*F. nucleatum* ^∗^	8 (19.05%)	22 (59.46%)	43 (71.67%)
*pks^+^ E. coli* ^∗^	9 (21.43%)	25 (67.57%)	42 (70.00%)
FOBT^∗^	0	24 (64.86%)	52 (86.67%)

WBC (10^9^/l), PLT (10^9^/l), RBC (10^12^/l), Hb (g/l), CEA (ng/ml), and CA19-9 (ng/ml). ^∗^Number of positive samples (percentage).

**Table 3:** Clinical model and biomarker outcome prediction of CRC, CAP, and controls.

Test result variable(s)	AUC (95% CI)	*P* value	Youden	Cut-off point	SEN (%)	SPE (%)	PPV	NPV	LR+	LR-
*CRC vs. control*										
CEA	0.826 (0.73, 0.91)	<0.01	0.506	2.25	71.4	79.2	0.70	0.80	3.4	0.3
CA199	0.627 (0.51, 0.75)	0.051	0.194	5.52	57.1	62.3	0.56	0.64	1.5	0.6
*F. nucleatum*	0.735 (0.59, 0.87)	<0.01	0.431	1.13^∗^	69.2	73.9	0.67	0.75	2.6	0.4
pks	0.810 (0.67, 0.96)	<0.001	0.666	2.25^∗^	93.3	73.3	0.90	0.80	3.5	0.1
Panel 1	0.855 (0.72, 0.98)	<0.001	0.645	-	75.0	89.5	0.75	0.89	7.1	0.2
Panel 2	0.859 (0.63, 1.0)	<0.001	0.629	-	80.0	82.9	0.78	0.84	4.6	0.2
Panel 3	0.844 (0.71, 0.97)	<0.001	0.670	-	84.6	82.4	0.82	0.85	4.8	0.1
Panel 4	0.871 (0.66, 1.0)	<0.001	0.718	-	75.0	96.8	0.77	0.96	23.4	0.2
Panel 5	0.887 (0.68, 1.0)	<0.001	0.713	-	75.0	98.1	0.77	0.98	39.4	0.2
*CAP vs. control*										
CEA	0.710 (0.57, 0.84)	0.006	0.391	1.90	71.4	67.7	0.67	0.72	2.2	0.4
CA199	0.764 (0.64, 0.88)	<0.001	0.421	3.23	67.9	74.2	0.67	0.75	2.6	0.4
*F. nucleatum*	0.741 (0.56, 0.91)	0.025	0.361	1.04^∗^	70.9	65.2	0.66	0.70	2.0	0.4
pks	0.818 (0.64, 0.98)	0.003	0.659	1.97^∗^	90.9	75.0	0.88	0.81	3.6	0.1
Panel 1	0.837 (0.70, 1.0)	0.032	0.575	-	85.1	72.4	0.81	0.78	3.0	0.2
Panel 2	0.720 (0.49, 0.94)	0.002	0.364	-	50.0	86.4	0.60	0.81	3.6	0.5
Panel 3	0.827 (0.62, 0.97)	<0.001	0.607	-	85.7	75.0	0.82	0.80	3.4	0.1
Panel 4	0.841 (0.51, 1.0)	<0.001	0.590	-	66.7	92.3	0.70	0.91	8.6	0.3
Panel 5	0.846 (0.57, 1.0)	<0.001	0.570	-	66.7	90.3	0.70	0.89	6.8	0.3
*CRC vs. CAP*										
CEA	0.684 (0.51, 0.85)	0.05	0.329	2.05	90.3	42.9	0.69	0.76	1.5	0.2
CA199	0.440 (0.26, 0.61)	0.52	0.166	28.03	45.2	71.4	0.69	0.48	1.6	0.7
*F. nucleatum*	0.514 (0.33, 0.69)	0.88	0.071	3.59^∗^	64.5	42.9	0.61	0.46	1.1	0.1
pks	0.389 (0.21, 0.56)	0.23	-	2.67^∗^	48.4	42.9	0.54	0.37	0.8	1.2
Panel 1	0.486 (0.31, 0.66)	0.88	0.166	-	45.2	71.4	0.69	0.48	1.6	0.7
Panel 2	0.479 (0.30, 0.65)	0.82	0.166	-	45.2	71.4	0.69	0.48	1.6	0.7
Panel 3	0.560 (0.38, 0.73)	0.52	0.163	-	80.6	35.7	0.64	0.57	1.2	0.5
Panel 4	0.472 (0.31, 0.66)	0.92	0.205	-	41.9	78.6	0.33	0.73	1.9	0.7
Panel 5	0.488 (0.29, 0.64)	0.76	0.205	-	41.9	78.6	0.33	0.73	1.9	0.7

SPE: specificity; SEN: sensitivity; Youden: Youden index; LR: likelihood ratio OB: occult blood; ^∗^1∗10^−4^. Clinical model panel 1: *F. nucleatum*+CEA+CA199; panel 2: *pks^+^ E. coli*+CEA+CA199; panel 3: *F. nucleatum+pks^+^ E. coli*+FOBT; panel 4: *F. nucleatum*+*pks^+^ E. coli*+CEA+CA199; panel 5: *F. nucleatum*+*pks^+^ E. coli*+CEA+CA199+FOBT.

## Data Availability

The data used to support the findings of this study are available from the corresponding author upon request.

## Abbreviations

CRC: Colorectal cancer
CAP: Colorectal adenomatous polyposis
F. nucleatum: Fusobacterium nucleatum
pks: Polyketide synthase
ROC: Receiver operating characteristic curve
AUC: Area under the curve
PPV: Positive predictive value
NPV: Negative predictive value
CEA: Carcinoembryonic antigen
ANOVA: One-way analysis of variance.
